# Combination of Complement-Dependent Cytotoxicity and Relative Fluorescent Quantification of HLA Length Polymorphisms Facilitates the Detection of a Loss of Heterozygosity

**DOI:** 10.1155/2014/541345

**Published:** 2014-04-03

**Authors:** Klaus Witter, Roland Reibke, Marion Subklewe, Robert Zahn, Teresa Kauke, Karsten Spiekermann, Michael Spannagl, Johanna Tischer, Wolfgang Hiddemann, Andrea Dick

**Affiliations:** ^1^Laboratory of Immunogenetics, Department of Transfusion Medicine, Cell Therapeutic Agents and Hemostaseology, University Hospital of Munich, Ludwig Maximilians University, Max-Lebsche-Platz 32, 81377 Munich, Germany; ^2^Department of Internal Medicine III, University Hospital of Munich, Ludwig Maximilians University, Max-Lebsche-Platz 32, 81377 Munich, Germany

## Abstract

Loss of heterozygosity (LOH) is a common event in malignant cells. In this work we introduce a new approach to identify patients with loss of heterozygosity in the HLA region either at first diagnosis or after HLA mismatched allogeneic HSCT. Diagnosis of LOH requires a high purity of recipient target cells. FACS is time consuming and also frequently prevented by rather nonspecific or unknown immune phenotype. The approach for recipient cell enrichment is based on HLA targeted complement-dependent cytotoxicity (CDC). Relative fluorescent quantification (RFQ) analysis of HLA intron length polymorphisms then allows analysis of HLA heterozygosity. The approach is exemplified in recent clinical cases illustrating the detection of an acquired allele loss. As illustrated in one case with DPB1, distinct HLA loci in donor and patient were sufficient for both proof of donor cell removal and evaluation of allele loss in the patient's leukemic cells. Results were confirmed using HLA-B RFQ analysis and leukemia-associated aberrant immunophenotype (LAIP) based cell sort. Both results confirmed suspected loss of HLA heterozygosity. Our approach complements or substitutes for FACS-based cell enrichment; hence it may be further developed as novel routine diagnostic tool. This allows rapid recipient cell purification and testing for loss of HLA heterozygosity before and after allogeneic HSCT in easily accessible peripheral blood samples.

## 1. Introduction

The graft versus leukemia (GvL) reaction is a major component of the immunotherapy after allogeneic hematopoietic stem cell transplantation (HSCT). T cell-mediated immune responses depend on the antigen presentation by HLA molecules. After HLA haploidentical hematopoietic stem cell transplantation, the mismatched HLA molecules themselves become potent targets of the GvL reaction. Being in the center of any specific immune response, loss of antigen presentation is an appealing mechanism for immune escape. However, diminished HLA expression, as seen, for example, in infections with some herpes viruses, prompts the attack of natural killer (NK) cells. Therefore immune escape by altered HLA expression was assumed to be highly improbable in vivo [1, 2].

The acquired loss of heterozygosity (LOH), resulting in duplication of oncogenes or loss of tumor suppressor genes, is a well-established mechanism in tumor evolution [3, 4]. In copy number neutral loss (also referred to as acquired uniparental disomy, aUPD) of HLA heterozygosity, the mismatched set of HLA alleles is replaced by a set of identical HLA alleles [5]. Therefore the major target of T cell-mediated graft versus leukemia (GvL) response is neutralized, while concomitant NK cell activation is prevented. Copy number neutral loss of HLA heterozygosity was found in up to 30% of patients relapsed from acute leukemia after HLA haploidentical HSCT [5]. It therefore represents one of the most important immune escape mechanisms.

In addition, loss of HLA heterozygosity was also described prior to any treatment [4, 6–10] in malignant cells, which might be a serious confounder in a patient's HLA typing. Despite its vital importance, copy number neutral loss of heterozygosity is easily missed by standard methods.

Relative fluorescent quantification (RFQ) is a technique used in a variety of fragment analysis applications that require peak area comparisons across samples. Microsatellites or single nucleotide polymorphisms, aneuploidy assay, or the detection of large chromosomal deletions [11, 12] can be used to detect LOH. Here, we addressed the question whether the numbers of HLA alleles in leukemia cells were altered. Detection of copy number neutral LOH requires relatively pure target cells. However, for example, acute myeloid leukemia specimen consists of a mixture of healthy and malignant cells. Enrichment of target cells, for example, by fluorescence-activated cell sorting (FACS), is restricted to cases where a leukemia-associated aberrant immune phenotype (LAIP) is available [13]. To circumvent these restrictions and enable broad screening for loss of HLA alleles in the context of HSCT, we developed a donor HLA-specific complement-dependent cytotoxicity assay, allowing highly effective enrichment of host-derived cells from peripheral blood.

The applicability of this approach is outlined in three cases where testing for HLA-LOH should be mandatory.

## 2. Patients, Material, and Methods

### 2.1. DNA Isolation

The isolation of genomic DNA was performed using the GenoM6 automated DNA isolation system together with the EZ1 blood kit (350 *μ*L) (both Qiagen, Hilden, Germany).

### 2.2. HLA Typing

High-resolution HLA typing was performed by a homemade sequence-based typing (SBT) approach, as described previously [14, 15]. Low-resolution HLA typing was performed with Labtype (One Lambda, Canoga Park, CA, USA) reverse sequence-specific oligonucleotide hybridization (SSO). The SSO tests were run on a Luminex 200 Fluoroanalyzer (Tepnel, Manchester, UK) following the manufacturer's protocol. Additional SSP-based HLA typing was performed.

### 2.3. Chimerism Analysis

The evidence of chimerism was observed by means of sequence-specific primer amplification (SSP; Olerup, Sweden; data not shown). SSP kits were used according to the manufacturer's instructions.

### 2.4. Cell Separation Methods and the Depletion of Donor Cells

To direct the RFQ analysis to the tumor cells, there was the need to remove the paternal cells from the peripheral blood cell mixture. Therefore, we depleted the paternal cells in analogy to complement-dependent cytotoxicity testing (CDC). Antibody-mediated complement-dependent cytotoxicity (CDC) refers to the classical method of complement activation that is triggered by opsonization of cells by antigen-specific antibodies (e.g., HLA antibodies) resulting in target cell-specific lysis. CDC is used in the daily routine in our HLA lab, monitoring patients awaiting solid organ transplantation or HSCT. This method enables either cross matching between recipient and donor using T and B cells or detection and specification of HLA antibodies.

### 2.5. Depletion of Paternal Cells

**Figure 1:**
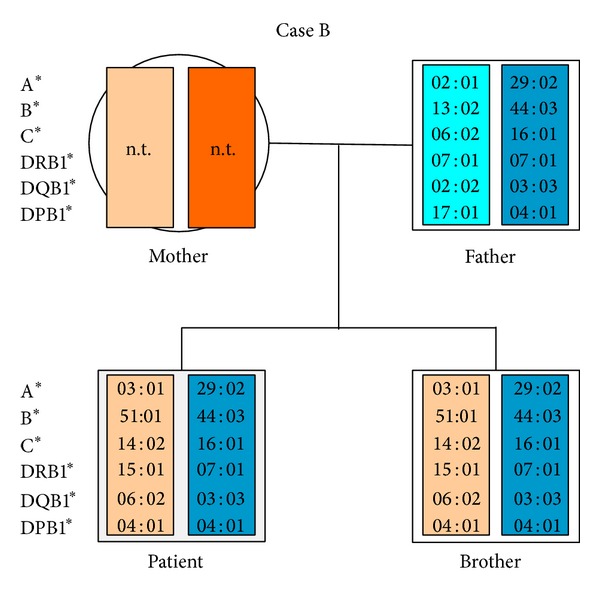
Pedigree of the second patient (Case B), showing HLA-typing of patient, brother, and father. As expected the patient and his father were HLA haploidentical. The mother could not be tested due to early death.

Peripheral blood mononuclear cells (PBMCs) were separated from whole blood by density gradient centrifugation. 4 mL of whole blood mixed with 3 mL of sodium chloride 0.9% solution was layered over 3 mL of FicollPaque (Biochrom, Berlin, Germany). The blood/Ficoll mixture was centrifuged for 10 min at 1500 g. Following centrifugation and washing, the PBMCs were resuspended in 500 *μ*L Hank's buffer. With respect to the HLA mismatches between the patient and his father (Figure 1), two monospecific antisera directed against the HLA of the paternal donor cells were utilized (see Section 2.6). 250 *μ*L of each antiserum, serum A containing anti-HLA-A2 antibodies and serum B containing anti-HLA-B13 antibodies, was incubated with 500 *μ*L of PBMCs at room temperature. After 30 minutes rabbit complement (Schriever, Bremerförde, Germany) was added and the sample was incubated for 1 h at room temperature. Before usage, each lot of complement was tested for specificity; there was cytotoxicity in the presence but not absence of specific HLA antibodies. To remove the lysed paternal cells, the cell suspension was then again layered over 3 mL of FicollPaque. After centrifugation at 1500 g the remaining cells were counted in a Neubauer hemocytometer and finally 350 *μ*L of the cell suspension was transferred to a sterile tube for DNA preparation.

### 2.6. Characterization of HLA Antisera

Patients awaiting solid organ transplantation or HSCT are routinely HLA-A, -B, -C, -DRB1, -DQB1, and -DPB1 typed and their sera are tested for the presence of HLA antibodies to prevent antibody-mediated rejection. Well-known immunizing events leading to HLA antibody production are pregnancy, blood or platelet transfusions, and previous organ transplantations. Due to the standards of EFI (European Federation for Immunogenetics, version 6.1.), HLA antibody testing of patients' sera is periodically performed and updated. Thus, a collection of ancient, well-characterized antisera from immunized patients is available in our lab. The two monospecific HLA sera used for the depletion of the paternal cells were derived from those patients. According to these EFI standards, sera are routinely screened for the presence of HLA classes I and II antibodies by CDC according to a standard protocol and by Luminex, a very sensitive solid phase-based method (One Lambda, Gene Probe, USA). This technology uses microbeads coated with class I or class II HLA antigens and a fluorescence flow analyzer. These methods are not only used for detection but also for specification of HLA class I and class II antibodies.

Thus, the A and B antisera used for donor cell depletion were well characterized by CDC and Luminex-based screening and specification for HLA antibodies. Both are monospecific for HLA-A2 and HLA-B13, respectively, without any detectable HLA class II antibodies.

### 2.7. Cell Separation by Immune Phenotype

In some patients, leukemic cells can be distinguished from healthy cells by an unusual expression pattern of surface antigens that is fairly exclusive to the patient's leukemic blasts [13, 16]. This leukemia-associated aberrant immune phenotypes (LAIP) allow identification and tracking of desired cells in multichannel fluorescence-activated cell sorting (FACS). The recurrent LAIP positive population showed a stable expression of CD34, CD33, and the lymphoid marker CD56. Applying a fluorescent-activated based cell sorting (FACS) we were able to accumulate leukemic blasts relatively exclusively. Approximately 50,000 CD34^+^/CD56^+^ cells were separated and DNA was isolated. Sorted CD34^−^/CD56^−^ cells served as controls.

### 2.8. Relative Fluorescent Quantification (RFQ)

We modified the microsatellite-based relative fluorescent quantification approach slightly and focused on the HLA gene length variations. In the majority of cases, virtually all of the short HLA-length polymorphisms (HLA-LP) in the relevant HLA loci are located in the intron sequences. This fact hinders heterozygous sequencing, but these circumstances allow us to discriminate between two or more alleles.

To obtain RFQ signals for the fragments, portions of differently HLA-typed alleles were amplified by PCR with the use of an AB 9700 thermocycler (Applied Biosystems (AB), Foster City, CA, USA) using NuLight547 or 6Fam 5′ fluorescent dye-labelled oligonucleotides (one of the amplification primers, Thermo Fisher Scientific GmbH, Ulm, Germany). The amplification products were analyzed by ethidium bromide-stained agarose gel electrophoresis. The PCR products were diluted 1 : 30 with HiDi formamide (AB), and, after the addition of 1 *μ*L of the GeneScan 500 ROX size standard, the PCR products were separated using an ABI Prism 310 Genetic Analyzer (AB). Analysis of the data was carried out using GeneMapper 5.0 (AB). All the samples were tested in triplicate.

To assess the sensitivity of our RFQ approach and to estimate the minimal required proportion of homozygous LOH bearing cells for detection, we set up a titration series (Table 1). The following two samples were mixed: genomic DNA of an HLA A*03/A*74 heterozygous individual was spiked with HLA-A*03 homozygous genomic DNA. The proportions of homozygous DNA resembled amounts of HLA-LOH bearing cells. Mixtures in different concentrations were tested for detection of allele imbalance by RFQ. As shown in Table 1, RFQ analysis was able to detect an imbalance in allele frequency even if >2/3 of the cells were heterozygous. Vice versa, the required proportion of target cells was >20–30% for reliable detection of LOH.

### 2.9. RFQ Analysis of DNA Samples

RFQ analysis was performed on DNA samples derived from the second patient's (Case B) peripheral blood cells, containing 34% blasts, the patient's peripheral blood cells after the CDC-based depletion of the paternal cells, the sorted CD34/CD56-positive bone marrow-derived cells and, as controls, the cells from the HLA-identical brother, and the patient's sorted CD34/CD56-negative bone marrow-derived cells.

### 2.10. Patient Characteristics

The patients' characteristics and cornerstones of treatment prior to diagnosis of HLA-LOH are summarized in Table 2.

## 3. Results

### 3.1. Detection of HLA-LOH after HLA Haploidentical HSCT (Case A)

A patient relapsed from acute myeloid leukemia (AML) after HLA haploidentical HSCT from his sister (Table 2). To ensure the relapse as host derived we performed an HLA retyping of a blood sample containing approximately 50% of leukemic blasts. In this situation, concerning the initial HLA typing, a mixed HLA chimerism indicated by B*08:01 (donor), B*13:02 (patient), and B*07:02 (both) (Table 3) would have been expected. However, the patient's original allele B*13:02 could not be detected by SBT. Since this could not be attributed to predominant donor chimerism, indicated by only diminished sequence signals for the donors specific HLA-B*08:01, an HLA-LOH in the leukemia cells was suspected. By LAIP based FACS we were able to enrich the leukemic cells and their DNA was HLA retyped by SBT. Consistent with the suspected loss of heterozygosity the leukemic blasts showed a complete homozygote HLA (HLA-A, B, C, DPB1, and DQB1) pattern with a loss of the mismatched HLA haplotype (Table 3). We therefore could prove an acquired loss of heterozygosity in the patient's relapsed leukemia cells.

### 3.2. Detection of HLA-LOH Utilizing HLA-Specific CDC (Case B)

A patient suffering from AML relapsed four years after allogeneic hematopoietic stem cell transplantation (HSCT) from his HLA-identical brother. Following multiple salvage therapies (Table 2), HLA haploidentical HSCT from his father was performed. However, despite additional immune stimulation with G-CSF and occurrence of chronic graft versus host disease (GvHD) the patient relapsed, with the relapsed disease being insensitive to further immune stimulation with IL-2 and natural killer cell infusion. This prompted testing for leukemia-specific immune escape mechanisms. The HLA types of father and son, shown in Figure 1, revealed the existence of several informative regions; for instance, HLA-A*03 could be distinguished from A*02 and A*29 and B*44 could be distinguished from B*13 and B*51 by length discrepancies (http://www.ebi.ac.uk/imgt/hla/align.html). Despite being informative, the allele relationship within the leukemic blasts was still masked by the donor, and, even after the depletion of the donor cells, it had to be proven that the cells had been completely removed. To address this issue, we analyzed three distinguishable HLA-DPB1 alleles present in the mixed cell population of recipient and donor. In spite of the homozygous HLA-DPB1 genotype of the patient, we were able to distinguish the donor- and host-derived cell populations, as both inherited DPB1*04:01 alleles and the DPB1*17:01 allele from the father showed different length in the microsatellite-like intron regions before exon 3.

**Figure 2:**
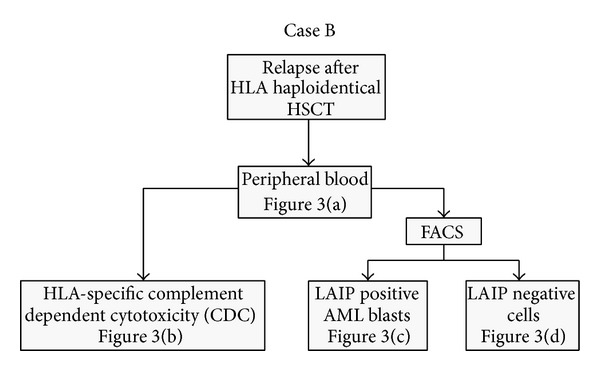
Flowchart showing the two alternative analytical approaches to a patient with suspected HLA-LOH. The corresponding results are illustrated in Figure 3 (Case B).

**Figure 3:**
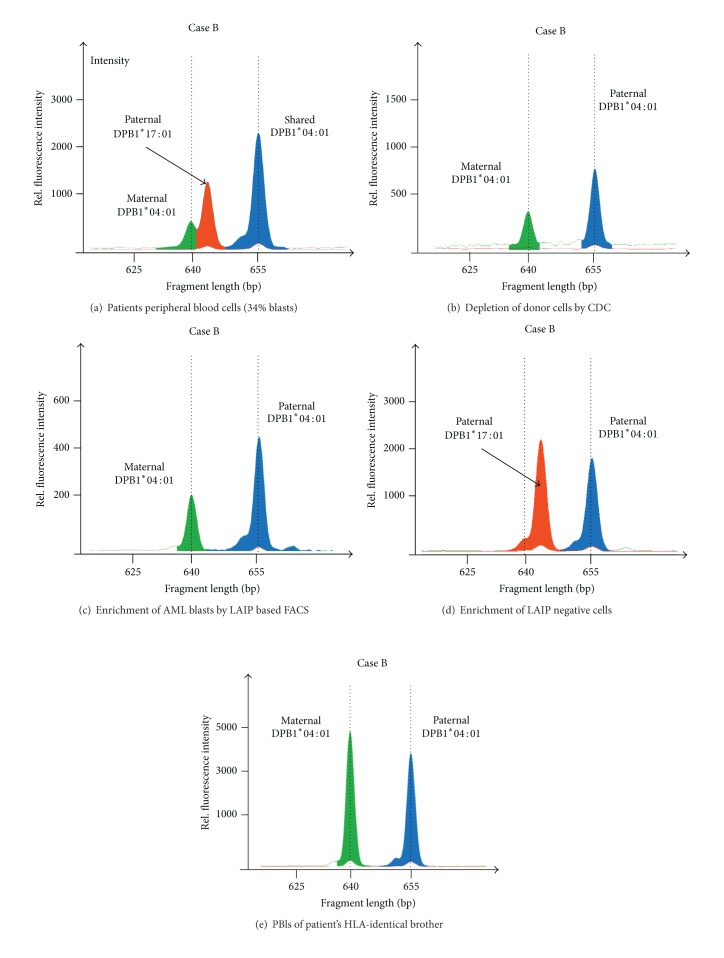
Relative fluorescent quantification (RFQ) of HLA-DPB1 (Case B). (a) RFQ analysis of the patient's sample showed a chimeric allele pattern resulting from donor (artificially colored in red) and host cells. (b) Complement-dependent cytotoxicity removal of the paternal cells by anti-HLA antibodies (anti-HLA-A2 and anti-HLA-B13) revealed an imbalance in the number of DPB1 alleles in the purified host-derived cells, latter enriched for leukemic cells. This result indicated that approximately 50% of the leukemia cells lacked the maternal (mismatched) DPB1*04:01 allele. Complete donor cell removal was proven by disappearance of DPB1*17:01. (c) Blast enrichment by LAIP based (CD34^+^/CD56^+^) FACS-sorted cells from a bone marrow aspirate confirmed the imbalance of the DPB1 alleles again assigned to the leukemic cells. No donor DNA was detectable. (d) RFQ analysis of LAIP negative (CD34^−^/CD56^−^) cells showed balanced DPB1 alleles. No maternal DPB1*04:01 was detectable. (e) As another control, the HLA-identical brother of the patient was tested, as expected a balanced number of alleles were detected.

**Figure 4:**
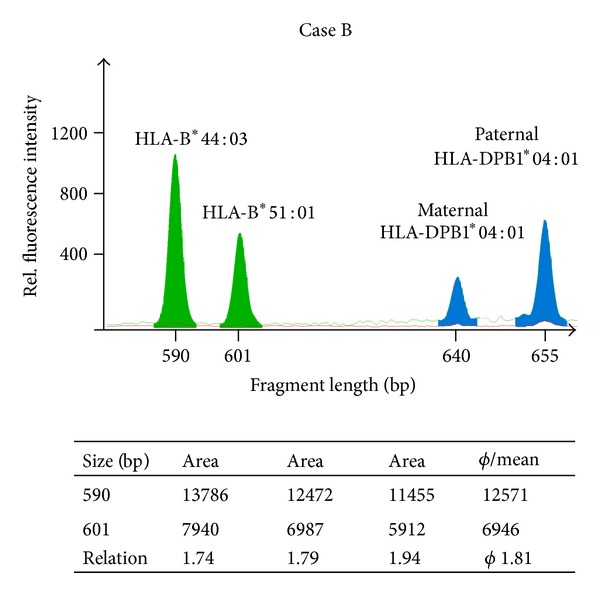
RFQ of HLA-B. Complement-dependent cytotoxicity donor-depleted cells of the second patient (Case B) were analyzed by RFQ which has taken advantage of an HLA-B length polymorphism in intron 4. The HLA-B fragments shown here (green filled peaks) have a size of 590 bp (B*44:03) and 601 bp (B*51:01). The fragments were amplified using the primers 5Bin3-389cons (TCC AGY ACT TCT GAG TCA CTT TAC) and 5′ Nulight547-labeled 3Bin4-56 (CCT GAC CCT GCT GAA GGG CTC C). The table below the figure shows the results of three independent experiments, which give an average allele measure of 1.82. These data are in good agreement with the HLA-DPB1 results. Nearly 50% of the leukemic cells also lacked the HLA-B region. Thus, both the HLA-DPB1 and the HLA-B locus revealed repression of the maternal-derived allele and HLA-B*51:01 was underrepresented. The HLA-DPB1 amplification products are also depicted (blue filled peaks).

To attribute the allele discrepancy to the patient's leukemic blasts we confirmed testing in separated cells (Figures 2–4).  Figure 3(a) shows the RFQ analysis of a blood sample containing a mixed cell population of donor and leukemic cells. This sample contained 34% of leukemic blasts and was drawn during the relapse period. The three peaks shown in the electropherogram resulted from the cells of the most recent stem cell donor, the father, (HLA type DPB1*04:01, 17:01) and from the patient, including the leukemic cells (HLA type DPB1*04:01, 04:01). The highest peak represents the shared DPB1*04:01 allele between the father and son and the peak in the middle represents the DPB1*17:01 allele unique to the father. The smallest peak resulted from the maternal DPB1*04:01 allele passed on to the patient, which harbors a slightly shorter microsatellite-like DNA sequence at the end of intron 2. Since the mixed cell population hampered the direct determination of the number of alleles in the patient's blasts, we eliminated the donor cells by CDC using HLA-A02- and HLA-B13-specific antisera directed against the unique class I alleles of the father. The surviving cells were harvested, and their DNA was analyzed by RFQ (Figure 3(b)). The electropherogram showed no evidence of the paternal DPB1*17:01 allele, indicating that the deletion of donor cells was complete and, conversely, that the remaining cells were exclusively host derived. The area under the curve ratio of the maternal and paternal DPB1*04:01 alleles was approximately 1 : 2; this imbalance indicated that the majority of host cells lost the mismatched HLA-DPB1 region.

To verify these results and to validate the specificity of the observed LOH, we utilized the patient's leukemia-associated immunophenotype (LAIP). By multichannel fluorescent-activated cell sorting (FACS) we aimed for the 7.5% LAIP positive cells from bone marrow; therefore, RFQ could be performed in well-defined cell populations. The RFQ analysis of the CD34^+^/CD56^+^ cells, resembling enriched leukemic blasts, confirmed the previously obtained allele ratio as depicted in Figure 3(c). Likewise, CD34^−^/CD56^−^ cells were donor derived (no maternal DPB1*04:01 allele) and showed equal peak heights (Figure 3(d)). As additional control, the patient's healthy HLA-identical brother was tested, showing as expected a balanced ratio between the DPB1 alleles (Figure 3(e)). In addition, further analysis of the CDC enriched cells confirmed a similar ratio for the HLA-B than for HLA-DPB1 region (Figure 4), proofing a broad imbalance in the allele number throughout the HLA region. Further RFQ analysis of the CDC donor-depleted cell sample showed a noninformative HLA-G [17] at the telomeric end of the HLA region (data not shown), indicating large homozygous regions spanning the entire HLA locus as described by Waterhouse et al. [18]. Since the degree of detected imbalance was clearly associated with accumulation of the leukemic blasts, we suspected a leukemia-specific loss of HLA alleles. 

### 3.3. Detection of HLA-LOH Prior to Allogeneic HSCT (Case C)

**Figure 5:**
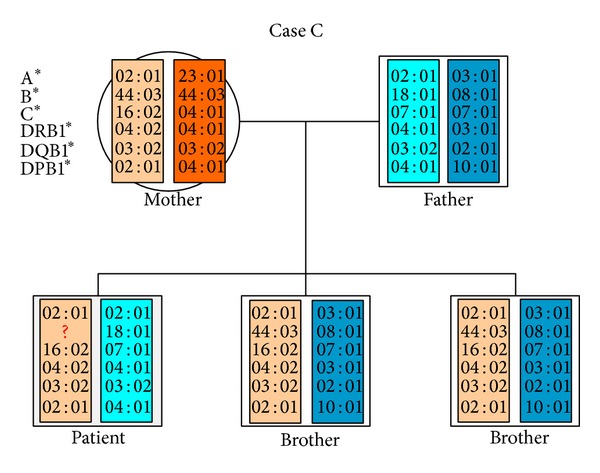
Pedigree of the third patient (Case C). As shown by family HLA typing and patient's decent he must be positive for HLA-B*44:03. Actual typing revealed a loss of this allele. This discrepancy was explained by very high blast counts in the tested blood sample.

**Figure 6:**
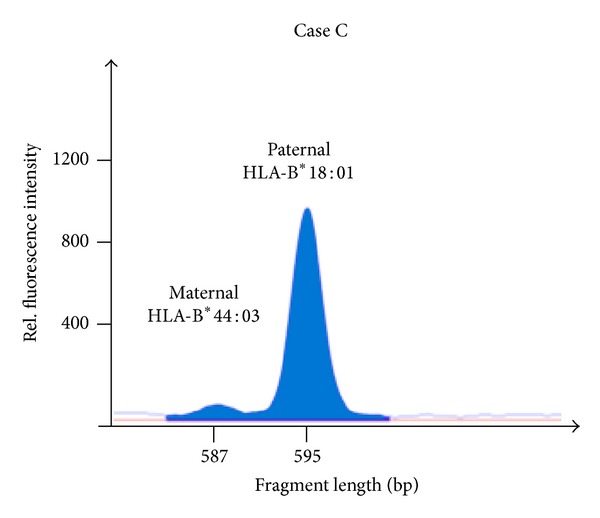
Allele loss in HLA-B visualized by RFQ (Case C). Due to the intron 4-length- polymorphism in HLA-B an amplicon of 595 bp (B*18:01) and 587 bp (B*44:03) was gained using the amplification primers 5Bin3-389cons (= 1319-42; TCC AGY ACT TCT GAG TCA CTT TAC) and 3Bin4-56 (= 1887-1908; CCT GAC CCT GCT GAA GGG CTC C). The HLA-B*44 allele is almost absent.

The third case illustrates the detection of a loss of HLA heterozygosity prior to any treatment (Table 2). A patient with acute lymphatic leukemia was HLA-typed for subsequent allogeneic HSCT. Standard HLA typing revealed a discrepancy in the patient's genotype. By family typing and according to patient's ancestry (Figure 5), the patient should have exhibited the HLA allele B*44:03. In contrast, the HLA-B*44:03 showed only a very weak signal in RFQ analysis (Figure 6), confirmed by SSO and SSP-based HLA typing (data not shown) and resembling a homozygous genotype. In retrospect, we were able to attribute this discrepancy to high blast counts (>80%) in the patients peripheral blood sample. We therefore assume a loss of HLA alleles (HLA-LOH) in this patient's leukemic cells prior to any treatment.

## 4. Discussion

The loss of HLA alleles is one of the most abundant resistance mechanisms after HLA haploidentical HSCT [5]. Its detection might hold significant implications for the patients' therapy and course. Therefore, testing for HLA-LOH should be mandatory in every patient relapsed after HLA haploidentical bone marrow transplantation. Furthermore its frequency and role in primary leukemogenesis are not clarified [10] and render it a pertinent source of error for HLA typing. Detection is often prevented by insufficient purity of target cells. However, alternative approaches, for example, next generation sequencing or real-time PCR are not only experimental but also expensive, time consuming and not always available or applicable.

The method presented herein is a robust and easy-to-use approach for testing in abundantly available peripheral blood samples. As presented, the selection of leukemic blasts by HLA-based CDC is reliable and the results are comparable to those achieved by LAIP based FACS. The availability of antisera against the majority of HLA-A and HLA-B antigens renders CDC to an optimal tool for cell enrichment and in combination with RFQ to an appropriate screening tool for HLA-LOH.

RFQ analysis of HLA alleles can be used to determine evidence of LOH. The loss of HLA alleles becomes evident by a shift in the ratio of the heterozygous alleles, approximating a homozygous genotype. Testing of various HLA and non-HLA gene loci in the MHC region allow to survey the extent of the LOH. Disparities between the patients' leukemic and healthy cells prove the acquired, leukemia-specific loss of mismatched “target” HLA alleles. 

Since not every allele is directly accessible for analysis, evidence may be derived from the neighboring genomic area as outlined in the second patient (Case B). Despite identical DPB1*04:01 alleles and therefore without immunological selection pressure, the DPB1 gene reflected a shift in the allele quantity caused by other HLA members in the nearby surrounding genomic area, such as the DQ, DR, or class I regions, which themselves were experimentally not directly accessible for quantification. This imbalance in the allele number throughout the HLA region was confirmed by the data for HLA-B. Specificity of HLA-LOH for leukemic cells was verified in well-characterized cell populations. With an increase of leukemic blast content the HLA-LOH became more evident in all tested samples. Conversely, in the controls, as, for example, the CD34^−^/CD56^−^ negative population, a normal HLA ratio was detected. The result of the CDC-based selection was equivalent to those achieved by leukemia-associated aberrant immune phenotype (LAIP) based FACS (Case B).

A detectable minor proportion of the heterozygous HLA genotype, even after LAIP based FACS, might be attributed to the inherently limited distinction of leukemic blasts from healthy cells. Even if a LAIP, mirroring the patient's relapses, is available, its specificity and sensitivity are limited [13, 16]. Indeed, the aberrant expression of CD56 may be seen in up to 4% of bone marrow-derived cells [16]. Furthermore, it is apparent that an acute leukemia consists of heterogeneous clones with various mutations [19]. Therefore, the LAIP might change or the HLA-LOH might account for immune escape only in some clones within the leukemic population.

By HLA-specific CDC, we were able to demonstrate complete removal of donor cells. A leukemia relapse, in contrast to rejection, is characterized by accumulation of leukemic, host-derived blasts. Therefore and despite accompanied regeneration of the “healthy” host-derived hematopoiesis [20], a sufficient purity for HLA-LOH detection is warranted. In addition, since healthy cells are targeted, this method is insensitive to mutations or phenotypic changes.

Furthermore, CDC might enable detection of HLA-LOH even in early stages and prior to allogeneic HSCT. HLA-LOH mutated cells are resistant to certain HLA-specific CDC, which might enable enrichment and testing of the desired cells.

On the other hand, since HLA testing is often performed at first diagnosis or progression of disease, the patients' samples often exhibit high blast contents, rendering them susceptible to actual “blast typing.” In accordance with other authors we identified pretransplant HLA-LOH as a pertinent source of error for the patients' HLA typing [4, 6–10]. As exemplified, the detection of primary HLA-LOH is tricky and might result in false HLA-typing. We therefore strongly recommend verification of any homozygous HLA typing by a leukemia free sample (e.g., buccal swap) or confirmatory family typing.

Due to the significance of HLA-LOH we intend to develop the presented approach into a routine screening method. Its implementation will assure optimal donor choice and hopefully better outcome after allogeneic HSCT.

## Figures and Tables

**Table 1:** Determination of LOH detection threshold by RFQ. Titrations series with artificial mixtures simulating (copy number neutral) loss of heterozygosity (LOH) in the HLA-A allele (for details see RFQ in material and methods).

Percentage of heterozygous DNA (A*74/A*03)	Percentage of homozygous DNA (A*03/A*03)	Assumed ratio of alleles A*74 : A*03	Detected peak area ratio A*74 : A*03
100%	0%	1 : 1,00	1 : 1,04
90%	10%	1 : 1,22	1 : 1,23
80%	20%	1 : 1,50	1 : 1,47
67%	33%	1 : 2,00	1 : 1,89
50%	50%	1 : 3,00	1 : 2,94

**Table 2:** 

	Case A	Case B	Case C
Diagnosis	t-AML*	AML	ALL
Age at first diagnosis	21	29	32
Date of first diagnosis	04/2007	05/2005	11/2010

Course of treatment			
Induction	Cytarabine + idarubicin/HAM	Sequential HAM	None
(1) Allogeneic HSCT	08/07: allogeneic HSCT (MUD)	07/05: allogeneic HSCT (MRD)	
	02/12: Relapse incl. chloroma/CNS	01/09: relapse	
Salvage therapies	Clofarabine	03/2009 Stem cell boost + GM-CSF	
		07/09 Relapse incl. chloroma	
		Cytarabine + gemtuzumab/ozogamicin	
(2) Allogeneic HSCT	05/12: HLA haploidentical HSCT (sister)	08/09: HLA haploidentical HSCT + G-CSF (father)	
Status at testing	03/13: Relapse	07/10: Relapse incl. chloroma, immunotherapy	First diagnosis

*Therapy related AML after 8x BEACOPP for Hodgkin's disease.

Abbreviations: AML: acute myeloid leukemia, ALL: acute lymphoblastic leukemia, MUD: matched unrelated donor, MRD: matched related donor, G-CSF/GM-CSF: granulocyte/granulocyte macrophage colony-stimulating factor, HAM: high dosed cytarabin/mitoxantron, CNS: central nervous system involvement.

**Table 3:** HLA class I and class II typing before and after HSCT (Case A).

HLA class I	A*-1	A*-2	B*-1	B*-2	C*-1	C*-2
Patient before Tx	03 : 01	24 : 02	07 : 02	13 : 02	06 : 02	07 : 02
Sister	03 : 01	24 : 02	07 : 02	08 : 01	07 : 01	07 : 02
Patient (pB) after Tx	*03 : 01 *	*24 : 02 *	*07 : 02 *	*08 : 01 *	*07 : 01 *	*07 : 02 *
LAIP pos. cells	03 : 01		07 : 02			07 : 02

HLA class II	DRB1*-1	DRB1*-2	DQB1*-1	DQB1*-2	DPB1*-1	DPB1*-2

Patient before Tx	01 : 01	07 : 01	02 : 02	05 : 01	n.d.	n.d.
Sister	01 : 01	03 : 01	02 : 01	05 : 01	n.d.	n.d.
Patient (pB) after Tx	*01 : 01 *	*03 : 01 *	*02 : 01 *	*05 : 01 *	*04 : 01 *	*04 : 01 *
LAIP pos. cells	01 : 01			05 : 01	04 : 01	

Abbreviations: pB: peripheral blood; LAIP: leukemia-associated aberrant immunophenotypes; n.d.: no data.
